# On the Use of NaOH Solution to Simulate the Crevice Conditions of a Nuclear Steam Generator

**DOI:** 10.3390/ma15238471

**Published:** 2022-11-28

**Authors:** Do-Haeng Hur, Geun-Dong Song, Jeoh Han, Soon-Hyeok Jeon

**Affiliations:** 1 Materials Safety Technology Development Division, Korea Atomic Energy Research Institute, Daejeon 34057, Republic of Korea; 2 Nuclear Materials Research Group, FNC Technology Co., Ltd., Yongin 17084, Republic of Korea

**Keywords:** sodium hydroxide, crevice environment, impurity concentration, magnetite deposit, steam generator, potential-pH diagram

## Abstract

The corrosion behavior and integrity of steam generator (SG) tube materials have frequently been tested in solutions containing sodium hydroxide (NaOH), assuming that NaOH is a typical contaminant concentrated in the crevices of SGs in a pressurized water reactor. The purpose of this study was to investigate the adequacy of using concentrated NaOH solutions to simulate the crevice environments of SGs. The dissolution behavior of magnetite deposit flakes formed in an operating SG was tested in a 0.4 wt.% NaOH solution at 300 °C, and the thermodynamic stability of magnetite was investigated using the potential-pH diagram for an iron–water system. The magnetite deposits were rapidly dissolved in the test solution, which was supported by the fact that magnetite is thermodynamically unstable under the test condition to dissolve to dihypoferrite ions (HFeO_2_^−^). These results indicate that research data obtained from concentrated NaOH solutions are not appropriate to apply to the crevice environments of SGs.

## 1. Introduction

**Figure 1:**
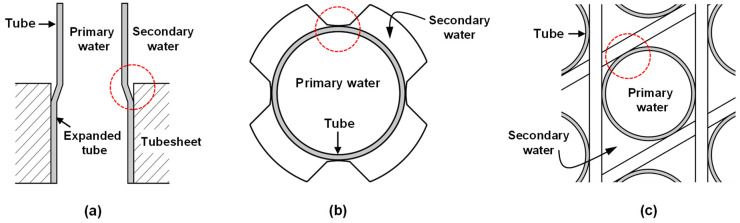
Typical crevice geometries around SG tubing at: (**a**) top of tubesheet, (**b**) broached quatrefoil-type TSP and (**c**) egg crate-type TSP. Crevices between tubing and TSP were denoted by the dotted red circles.

In a pressurized water reactor, high-pressure steam to drive a turbine is generated by a classic shell-and-tube type steam generator (SG). Both end sides of a heat-transfer tube in the SG are leak-tight expanded in a thick tubesheet. Tube bundles are supported with horizontal and vertical plates to prevent flow-induced vibration and fretting wear of the tubing. Therefore, these manufacturing processes inevitably create numerous crevices around tubes expanded in the tubesheet and tubes supported by the tube support plates (TSPs). Typical crevice geometries around SG tubing are shown in Figure 1.

It is well known that aggressive impurity chemicals in the SG feedwater are concentrated into the narrow-heated crevices, thereby inducing corrosion damage of the SG tubing including intergranular attack, pitting, denting, and stress corrosion cracking. On the other hand, corrosion products released due to the flow-accelerated corrosion of iron-based piping materials are transported into the SGs and deposited on the surfaces of the SG materials including tubes, TSPs, and tubesheets. The SG deposits are mainly composed of magnetite (Fe_3_O_4_) that contains numerous pores [1,2,3,4,5,6,7]. As a result, chemical impurities are easily concentrated within the deposits. Therefore, impurity concentration and the resultant corrosion damage are accelerated in the crevices covered with the porous deposits [8,9,10].

SG water chemistry is controlled on the all-volatile treatment basis using a pH control agent (ammonia, ethanolamine, morpholine, etc.) and an oxygen scavenger (hydrazine) [11]. The pH of the SG feedwater is normally adjusted in the range of 9.2 to 10.0 at 25 °C to minimize flow-accelerated corrosion and the resultant transport of corrosion products to the SGs [12]. Bulk water impurities in the operation of SGs are strictly limited as follows: sodium < 5 ppb, chloride < 10 ppb, and sulfate < 10 ppb [11]. Nevertheless, the bulk water can transiently be contaminated by some sources: seawater influx due to condenser leakage, unwanted discharge from ion exchange resins, and impurity ingress during maintenance processes.

Against this background, corrosion tests for SG materials have been performed in aqueous solutions containing various contaminants to simulate corrosive crevice environments. The typical contaminants are as follows: sodium, chloride, sulfur, lead, silica, etc. [9,10,13,14,15,16]. Accordingly, the following solutions have been widely used: solutions containing sodium hydroxide (NaOH) [17,18,19,20,21,22], sodium chloride (NaCl) [23,24,25], sulfate (SO_4_^2−^), sulfite (SO_3_^2−^), tetrathionate (S_4_O_6_^2−^) and thiosulfate (S_2_O_3_^2−^) [26,27,28,29], lead oxide (PbO) [30,31,32], and silicone compounds (SiO_2_, Na_2_SiO_3_) [33,34,35]. Among them, NaOH solution has been frequently used to investigate the performance of SG tubing under impurity-concentrated crevice conditions. As of now, more than 180 papers have been published in journals listed in the Science Citation Index, since the 1970s. The concentration of NaOH in the test solutions was up to 50 wt.%. It should be emphasized that accelerated corrosion experiments should be relevant to the application [36]. In other words, if the water chemistry used in a corrosion test is not suitable, the test results are useless for application to SG crevice conditions. In this context, the purpose of this study was to investigate the adequacy of using concentrated NaOH solutions to simulate the crevice environments of SGs. To this end, this study examined the immersion corrosion behavior of deposit flakes taken from an operating SG in 0.4 wt.% NaOH solution at 300 °C. The thermodynamic stability of magnetite was also investigated at various NaOH concentrations.

## 2. Materials and Methods

The corrosion test of SG deposits was performed using deposit flakes taken from an operating nuclear SG. The deposit flakes were pulled out from the outer surfaces of SG tubes during sludge lancing after operation of fuel cycle 27 in a pressurized water reactor. The SGs of the plant are equipped with nickel-based Alloy 600 tubes (15Cr-9Fe-76Ni in wt.%) as the heat-transfer tubing and have operated under a reducing all-volatile treatment condition with a pH range of 9.0 to 9.6 since the first commercial operation. The deposits were mostly composed of magnetite and contained small amounts of trevorite (NiFe_2_O_4_), jacobsite (MnFe_2_O_4_), and metallic copper. The porosity of the deposits was measured to be approximately 9.8%. The detailed characteristics of the deposits are given elsewhere [1].

**Figure 2:**
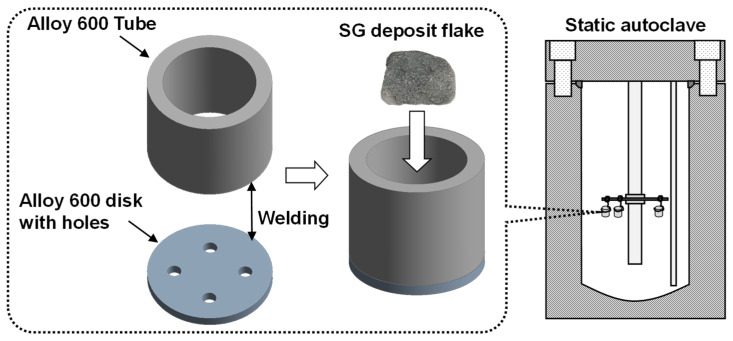
A schematic of an apparatus used for the dissolution test of SG deposit flakes.

The dissolution behavior of the deposit flakes was investigated under two different water chemistry conditions listed in Table 1. The reference condition simulates a normal bulk water chemistry with a pH_25°C_ of 9.5. The reference solution was prepared by adding a dilute NaOH solution to deionized water, resulting in a NaOH concentration of 1.075 × 10^−4^ wt.% and a pH of 9.5 at 25 °C. The caustic condition represents a simulated crevice environment of an SG, which contains 0.4 wt.% NaOH. As shown in Figure 2, an SG deposit flake was placed in an Alloy 600 container, of which the top end was open and the bottom sheet was pierced, thereby facilitating the interaction of the flake with the solution. The tests were conducted with the respective chemistry conditions using two separate static nickel autoclaves with a 2 L capacity. One liter of the test solution was loaded into the autoclave, de-aerated by bubbling argon gas (a purity of 99.999%) into the solution after the closure of the autoclave at a rate of 300 mL/min at room temperature (~23 °C) for 3 h and then heated to 300 °C. It was confirmed through a separate experiment before the immersion test that dissolved oxygen was removed below 10 ppb by the de-aeration process. The tests were interrupted to examine the morphology change of the flake samples after 20 and 30 days. An optical microscope (Keyence, Osaka, Japan, model VHX-5000) and a scanning electron microscope (SEM, TESCAN, Brno, Czech Republic, model LYRA3) were used to observe the morphology of the samples. SEM micrographs were taken at an accelerating voltage of 5 kV. The test solution was refreshed after each examination.

## 3. Results and Discussion

### 3.1. Dissolution Behavior of the SG Deposit Flakes

**Figure 3:**
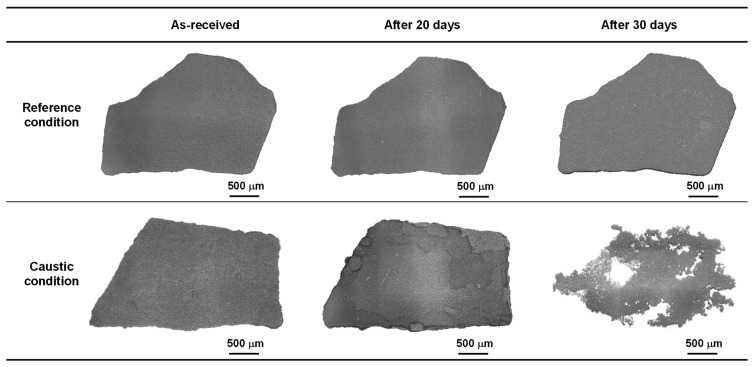
Optical micrographs showing the change in the morphology of the SG deposit flake samples exposed to the two different water chemistry conditions at 300 °C.

Figure 3 shows the optical images of the SG deposit flake samples exposed to the two different water chemistry conditions at 300 °C. In the reference solution, there was no change in the surface morphology of the sample up to 30 days. However, it was difficult to recognize the original shape of the deposit sample immersed in the caustic solution. In other words, the sample was rapidly dissolved and only a certain amount of the flake was left after the 30-day immersion.

**Figure 4:**
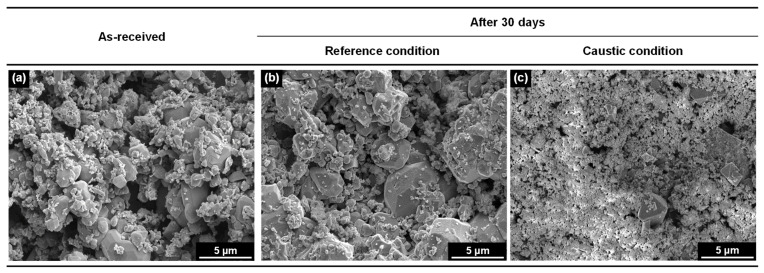
SEM micrographs showing the surface morphology of the SG deposit samples exposed to the two different water chemistry conditions at 300 °C for 30 days: (**a**) as-received, (**b**) immersed in the reference solution for 30 days, and (**c**) immersed in the 0.4 wt.% NaOH solution for 30 days.

The surface morphologies of the samples exposed to the solutions were further examined using SEM. The as-received deposit flakes were hard and brittle in nature. As shown in Figure 4a, the deposit flakes were typically composed of numerous magnetite particles ranging from tens of nanometers to several micrometers in size. The particles were polyhedral or roundish in shape. More detailed morphology, microstructure, and chemical composition of the deposits are given elsewhere [1]. As shown in Figure 4a,b, the size and shape of the particles of the deposit flakes did not change after exposure in the reference solution for 30 days. However, comparing Figure 4c with Figure 4a, the size of the particles was significantly decreased, and the shape tended to have more rounded edges, indicating that the deposit particles were dissolved in the caustic solution.

### 3.2. Thermodynamics for the Dissolution of the SG Deposit Flakes

**Figure 5:**
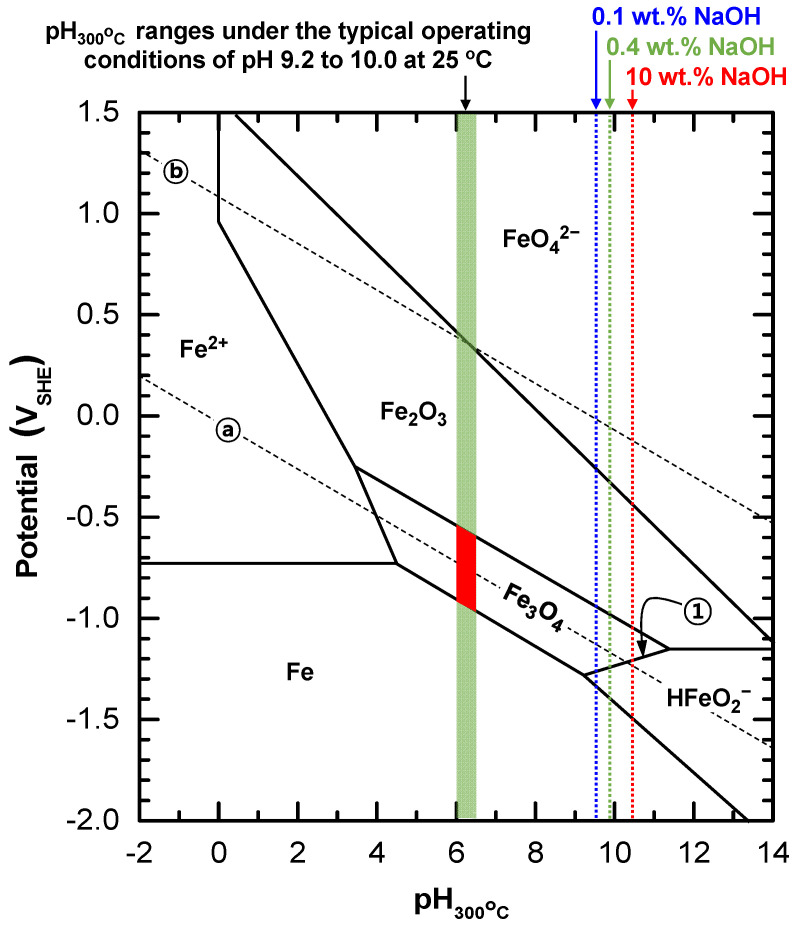
Potential-pH diagram for the iron–water system at 300 °C [37]. pH values at 300 °C in various environments were calculated using the MULTEQ code (Version 4.2.0) and superimposed in the diagram. Lines ⓐ and ⓑ represent respectively the reduction equilibrium of water according to the reaction H_2_ = 2H^+^ + 2e^−^ and the oxidation equilibrium of water according to the reaction 2H_2_O = O_2_ + 4H^+^ + 4e^−^ at a hydrogen or oxygen pressure of 1 atm. Line ① expresses the equilibrium of Reaction (1), 3HFeO_2_^−^ + H^+^ = Fe_3_O_4_ + 2H_2_O + 2e^−^.

The dissolution of the SG deposits indicates that the deposits are thermodynamically unstable in 0.4 wt.% NaOH solution. The deposits can be assumed to be magnetite because they are mostly composed of magnetite [1]. Therefore, the stability of the magnetite deposits can be inferred using a potential-pH diagram. Figure 5 shows the potential-pH equilibrium diagram for an iron–water system at 300 °C [37]. pH values at 300 °C in various environments were calculated using the MULTEQ code (Version 4.2.0) and presented in the diagram. Typical operating pH values of 9.2 to 10.0 at 25 °C were in the pH range of 6.0 to 6.5 at 300 °C, which are denoted by the green rectangle. pH_300°C_ values with NaOH concentration were calculated to be 9.5 at 0.1 wt.% NaOH solution, 9.8 at 0.4 wt.% NaOH solution, and 10.4 at 10 wt.% NaOH solution, which are presented by the dotted vertical lines. It can be deduced that the SG magnetite deposits used in this study were formed within the stable domain of magnetite, which is shown in the red box. Similarly, the lack of dissolution of the deposit flakes immersed in the reference solution indicates that the deposits are still located in the stable area of magnetite during the immersion tests. Therefore, based on the immersion test result and the diagram, the equilibrium potentials of magnetite are thought to be situated close to the hydrogen line in the typical operating pH_25°C_ ranges of 9.2 to 10.0.

Let us consider the following electrochemical reaction:

3HFeO_2_^−^ + H^+^ = Fe_3_O_4_ + 2H_2_O + 2e^−^ (1)

The equilibrium of this reaction is expressed by line ① in Figure 5. The standard equilibrium potential ($E^{o}$) of Reaction (1) at 25 °C is described as follows:

(2) $$E^{o}=\frac{\mu_{Fe_{3}O_{4}}^{o}+2\mu_{H_{2}O}^{o}-3\mu_{HFeO_{2}^{-}}^{o}\;}{2F}$$

where $\mu_{i}^{o}$ is the standard chemical potential of the *i* substance, and *F* is the Faraday constant (96,485 Cmol^−1^). Based on the following values of the standard chemical potentials ($\mu_{Fe_{3}O_{4}\;}^{o}$= −242,400 cal, $\mu_{H_{2}O}^{o}$ = −56,690 cal, and $\mu_{HFeO_{2}^{-}}^{o}$ = −90,627 cal) [38], $E^{o}$ is calculated to be −1.819 V_SHE_ from Equation (2). Now, the equilibrium potential (E) of Reaction (1) at 300 °C can be calculated using the following Nernst equation:

(3) $$E=E^{o}+2.3\frac{RT}{2F}\log\frac{(a_{Fe_{3}O_{4}}){\left(a_{H_{2}O}\right)}^{2}}{{\left(a_{HFeO_{2}^{-}}\right)}^{3}\left(a_{H^{+}}\right)}\;$$

(4) $$E=-1.819+0.0568\mathrm{pH}-0.1706\log a_{HFeO_{2}^{-}}\;$$

where *R* is the gas constant (1.987 calmol^−1^K^−1^), *T* is the absolute temperature (K), and $a_{i}$ is the activity of the *i* substance. For the 0.4 wt.% NaOH solution, since magnetite is dissolved, the potentials at which the HFeO_2_^−^ species (dihypoferrite ion) is stable are in the range of −1.239 to −1.395 V_SHE_, shown in Figure 5. From Equation (4), these values correspond to an $a_{HFeO_{2}^{-}}$ of 0.73 to 6.0. Consequently, these calculations provide evidence that magnetite is thermodynamically unstable in 0.4 wt.% NaOH solution, leading to a dissolution to HFeO_2_^−^ ions. This is consistent with the immersion test result showing a rapid dissolution of magnetite in 0.4 wt.% NaOH solution. In a similar manner, we can predict that magnetite is no more stable in a caustic solution containing NaOH over 0.1 wt.% under the reducing environments of SGs.

Based on this work, if caustic environments are formed in heated crevices covered with magnetite deposits and within porous deposits on the tubes, the deposits would be dissolved. In contrast, the deposit inventory of SGs increases during plant operation and is thus removed by sludge lancing and chemical cleaning [39,40,41]. The deposit flakes used in the dissolution test were also formed in an operating SG. Therefore, the results obtained in this work indicate that concentrated NaOH solutions are not relevant to the real crevice environments of SGs.

Corio showed for the first time in the world that intergranular corrosion and stress corrosion cracking of the Alloy 600 SG tube material can occur in deoxygenated pure water [42], while Copson failed to reproduce such attack [43,44]. The essential reason is that Copson used boiling MgCl_2_ solutions as accelerated test conditions, which are not relevant to nuclear environments. Similarly, test results in concentrated NaOH solutions may be misleading and not suitable for evaluating the corrosion behavior of SG tubing materials. Consequently, the test parameters should be chosen to closely simulate the conditions encountered in the SG crevices during operation.

## 4. Conclusions

This study investigated the stability of magnetite deposits through immersion experiments in normal and caustic solutions and thermodynamic consideration for an iron–water system at 300 °C. The magnetite deposits formed in a real SG were rapidly dissolved in 0.4 wt.% NaOH solution. This result is consistent with the thermodynamic calculation that magnetite is not stable in a solution containing NaOH over 0.1 wt.% dissolving magnetite to dihypoferrite ions. These results indicate that concentrated NaOH conditions are not applicable in simulating the real crevice environments of SGs. Therefore, the research results from tests conducted in concentrated NaOH solutions may be misleading and not suitable for evaluating the corrosion behavior of SG tubing materials.

## Figures and Tables

**Table 1:** Experimental conditions for the magnetite dissolution tests.

Test Condition	NaOH Concentration (wt.%)	pH at 25 °C	Temperature (°C)	Test Duration (Day)
Reference condition	1.075 × 10^−4^	9.5	300	20, 30
Caustic condition	0.400	13.1		

## Data Availability

The data are not publicly available due to legal or ethical reasons.
